# Design and simulation of a solar array deployment mechanism for a small satellite using implicit time-stepping

**DOI:** 10.1038/s41598-026-37568-x

**Published:** 2026-02-17

**Authors:** George B. Saad, Ahmed R. Desoki, Mohamed Kassab

**Affiliations:** 1 Egyptian Space Agency, New Cairo, 11865 Egypt; 2 Aerospace Engineering Department, Cairo University, Giza, 12613 Egypt

**Keywords:** Implicit analysis, Convergence stability, Transient analysis, Finite element analysis, Damping effect, Nonlinear analysis, Robust convergence, Engineering, Mathematics and computing

## Abstract

Solar Arrays (SA’s) of a satellite are typically folded within the launcher. After the satellite is inserted into its orbit, SA’s are unfolded (or deployed) and locked. The deployment is a critical operation as its failure translates to catastrophic satellite mission failure. Therefore, the design of the SA Deployment Mechanism (DM) must be robust. The design must additionally ensure smooth deployment and gentle locking not to damage the SA’s nor the satellite structure. This work demonstrates designing a SADM having reasonable deployment speed yet smooth locking. This design is verified by simulation using transient Finite Element Analysis (FEA) employing implicit time-stepping scheme. Deployment large rotations, complex contacts and rapid locking, however, caused challenging convergence difficulties. This work demonstrates design, optimization, simulation and difficulties overcoming for a typical SADM. This also extends to any relatively slow mechanism.

## Introduction

Solar Arrays (SA) are the source of power supply of a satellite. The span of solar arrays can be too large, many times larger than the satellite dimensions. Therefore, these arrays are typically folded while the satellite is mounted on the launcher. This is a must because of the limited space of the launcher fairing, and due to the launcher stiffness requirements. After the satellite is inserted into its designated orbit, the solar arrays are to be unfolded, or deployed. Upon completion of deployment, locking must be ensured so that the arrays do not fold again. The system that is responsible for deployment and locking is denoted as the SA Deployment Mechanism (DM). The SADM is a critical system in the satellite as its failure translates to catastrophic mission failure. Therefore, the design of the SA Deployment Mechanism (DM) must be robust.

Due to the large dimensions of the SA’s and fragility of the solar arrays, the deployment and locking must be reliable, slow, smooth and gentle not to damage the SA’s nor the satellite structure. The SADM should be designed to optimally satisfy these requirements, while remaining simple.

This work demonstrates an analytical model to design a typical SADM to achieve the aforementioned requirements. This simplified model is then verified by simulation using transient Finite Element Analysis (FEA) with implicit time-stepping. The large rotations, complex contacts and rapid locking of the DM however make the convergence too difficult. Therefore, an appropriate adaptive time-stepping algorithm is proposed to overcome convergence difficulties and obtain the solution. This work is extendible to any relatively slow mechanism.

The design concept is presented in Sect. “Design concept”. In Sect. “Analytical model of deployment”, the SADM is analytically modeled, and the appropriate model parameters are designed. In Sect. “Finite element analyses (FEA) of deployment”, a FE model of the SADM is established. The appropriate time stepping schemes are discussed and the implicit scheme is chosen as the appropriate scheme. The solution however failed to converge initially. Therefore, an appropriate adaptive time-stepping algorithm is discussed to overcome the convergence difficulties and successfully obtain the solution. In Sect. “Results and discussion”, the results are presented and discussed.

## Design concept

**Fig. 1:**
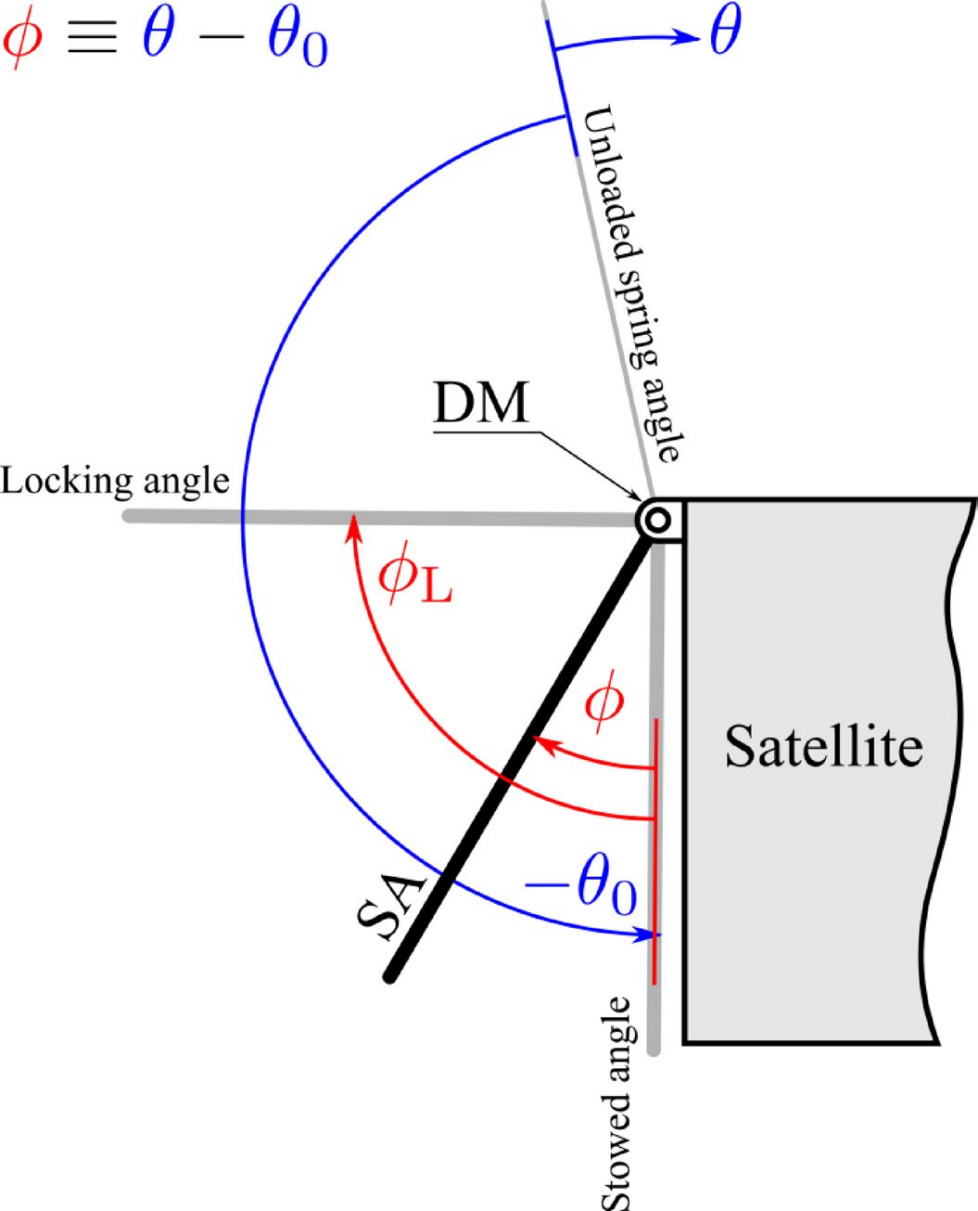
Operating angles of the SA.

Figure 1 shows the sign convention of operation positions of the SADM. The “Stowed angle” describes the folded position of the SA to the satellite body (initial position). The “Locking angle” describes the flight position of the SA w.r.t the satellite. The “Unloaded spring angle” describes the zero-moment position achieved by the torsional drive spring.

It is required to open the SA from the initial position to the flight position smoothly and safe with few seconds. In order to avoid reaching to maximum deflection (zero-moment position), it is designed a locking groove and pin to stop the deployment at the required angle (locking position).

**Fig. 2:**
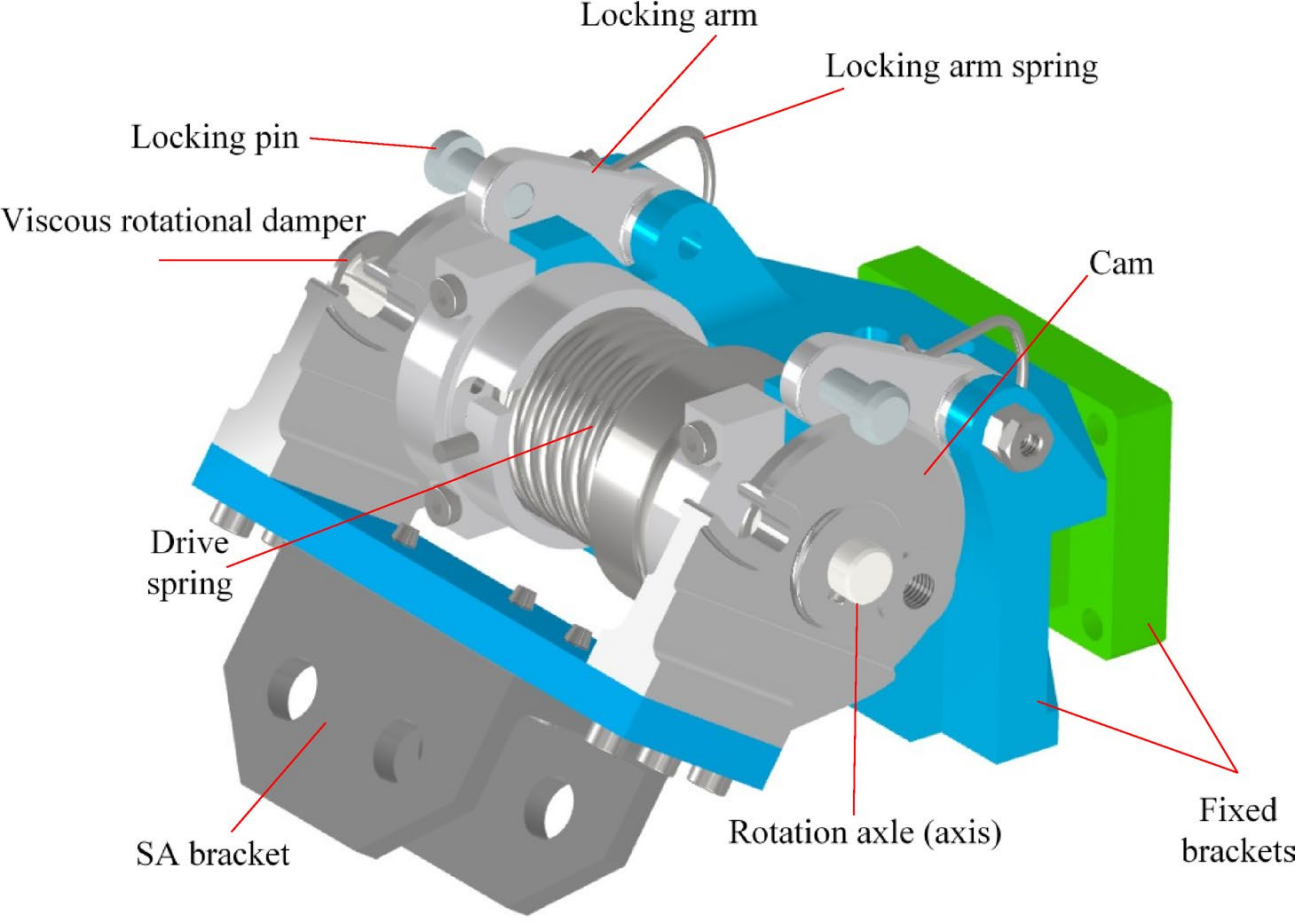
Details of the DM.

Figure 2 shows the components of the DM. The DM is composed of the following parts:

1. Fixed part attached to the satellite body.
2. Movable part attached to the SA.
3. Main drive source (torsion spring).
4. Operating cam joined with movable part.
5. Viscous rotational damper.
6. Locking arm spring.
7. Locking arm.
8. Locking pins.
9. Mounting screws.

Aluminum 7075-T6 is chosen for the most brackets while stainless steel is chosen for the locking pins.

**Fig. 3:**
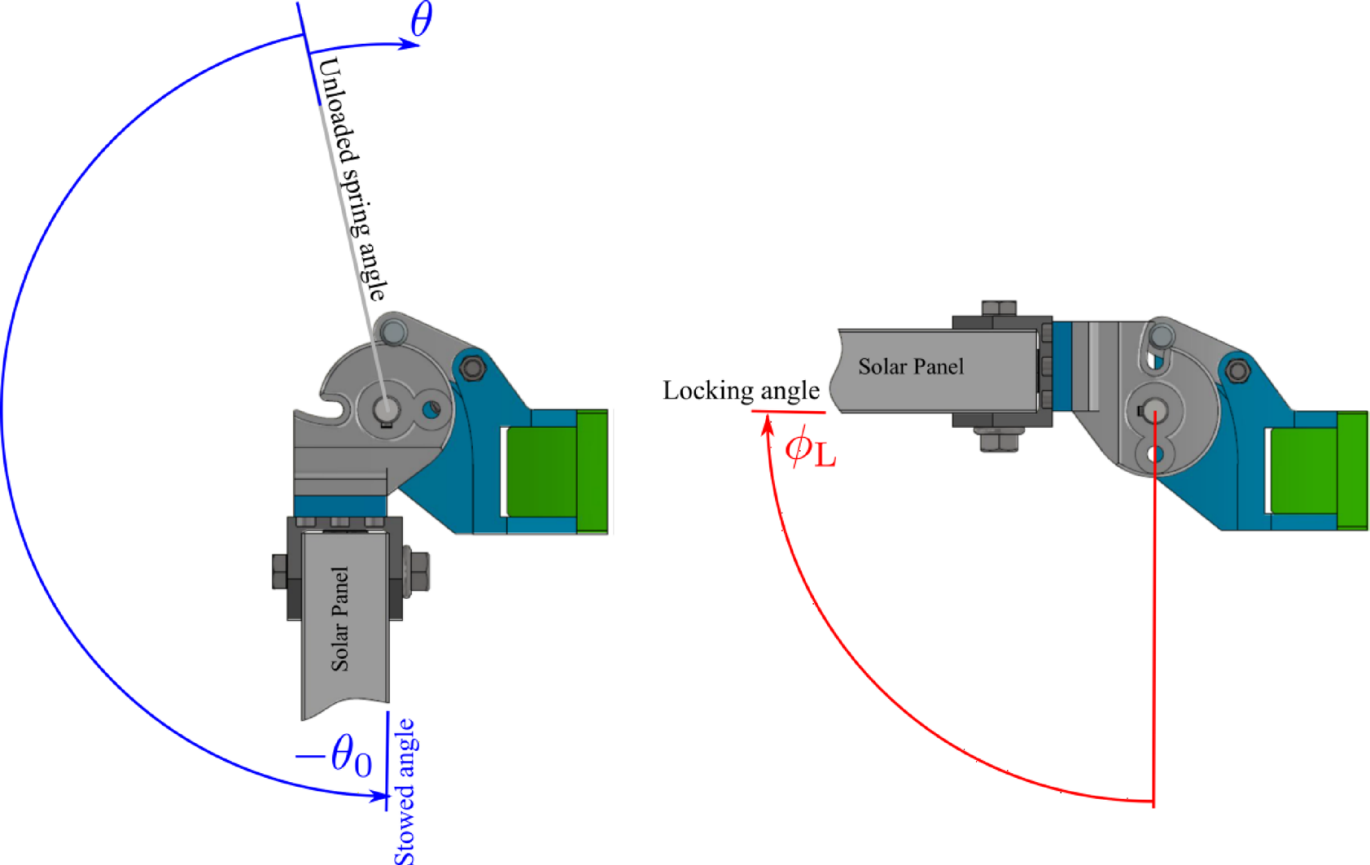
Side view of the DM.

After the satellite is inserted into its orbit, the DM is released to deploy the SA from the stowed angle at $\phi =0$ to locking angle at $\phi ={\phi}_{L}=9{0}^{^\circ }$. To initiate the deployment, the torsional drive spring is preloaded with the preload angle ${\theta}_{0}$. ${\theta}_{0}$ is chosen larger than the locking angle ${\theta }_{L}$ to assure that the SA reaches the locking angle with adequate torque. The angle $\phi$ is related to the angle $\theta$ as $\phi \equiv \theta -{\theta}_{0}$. Figure 3 shows a side view of the DM. As depicted in the figure, during the deployment, the locking pin is pushed in contact with the operating cam surface, till it drops into the locking groove, which ends the deployment.

To avoid sudden locking of the DM, the deployment angular velocity must be designed to decelerate before the instant of locking. This is explained in Sect. 3.

## Analytical model of deployment

**Fig. 4:**
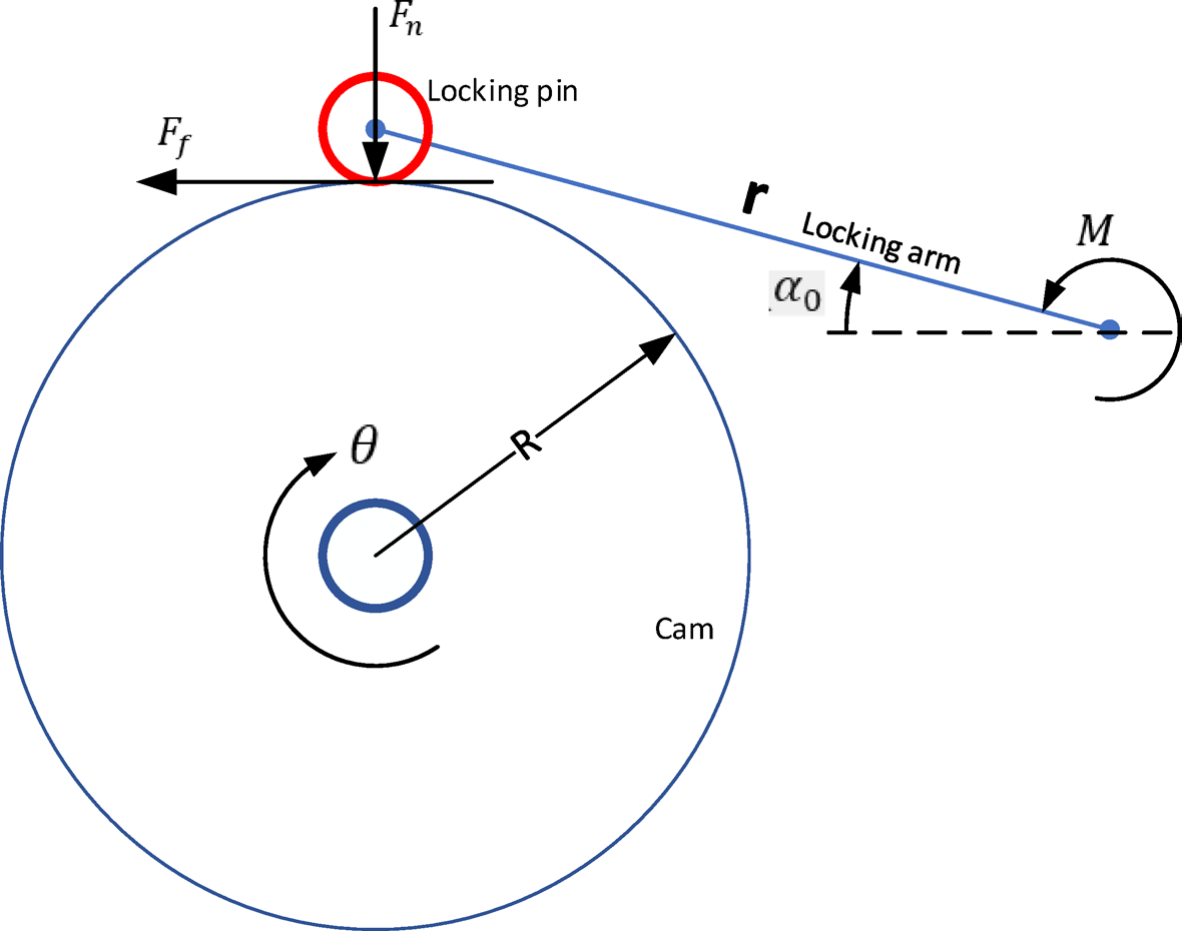
Free body diagram of the rotating part and locking pin.

Figure 4 shows the free body diagram of the operating cam and locking arm. From equilibrium of the locking arm, we can write

$$ {F}_{\mathrm{n}}=\frac{M}{r\ \mathrm{cos}({\alpha}_{0})}$$

where $$ {F}_{\mathrm{n}}$$ is the normal force of the locking pin acting on the operating cam, $$ M$$ is the preload torque in the locking arm spring.

The frictional force is thus calculated as

$$ {F}_{\mathrm{f}}=2\mu {F}_{\mathrm{n}}$$

where $\mu$ is the dynamic friction coefficient.

The rotational equation of motion is obtained from Lagrange’s equation. Let $\theta (t)$ denote the rotation of the deployment about its hinge as shown in Fig. 1. The kinetic energy $T$ and the elastic potential $U$ are

$$ T=\frac{1}{2}J{\dot{\theta }}^{2},     U=\frac{1}{2}k{\theta }^{2}$$

for linear stiffness $$ k$$. Non-conservative generalized work is represented by $$ \partial W/\partial q$$, which here collects viscous damping and the assumed constant locking (frictional) moment: $$ \partial W/\partial q=c\dot{\theta }-{M}_{f}$$, where $$ c$$ is the viscous damping coefficient and $$ {M}_{f}$$ is the constant resisting moment due to the locker and $$ q$$ is generalized coordinate = $$ \theta $$. Applying Lagrange’s equation

$$ \frac{\partial}{\partial t}\left(\frac{\partial T}{\partial \dot{q}}\right)+\frac{\partial U}{\partial q}=\frac{\partial W}{\partial q}$$

The equation of motion of the DM is systematically written as

1 $$ J\ddot{\theta }+c\dot{\theta }+k\theta (t)+{M}_{\mathrm{f}}=0$$

where $$ J$$ is the moment of inertia of the SA around the center of the cam, $$ c$$ is the damping constant of the viscous rotational damper at the cam center, $$ k$$ is the drive spring torsional stiffness and $$ {M}_{\mathrm{f}}$$ is the resistance moment due to friction.

$$\begin{aligned} {M}_{\mathrm{f}}&\equiv {F}_{\mathrm{f}}R\\& =2\mu {F}_{\mathrm{n}}R\\& =\frac{2\mu MR}{r \ \mathrm{cos}({\alpha}_{0})}\end{aligned}$$

Dividing both sides by $$ J$$ yields the non-dimensional form

$$ \ddot{\theta }+2\zeta {\omega}_{n}\dot{\theta }+{\omega}_{n}^{2}\theta (t)+\frac{{M}_{f}}{J}=0$$

Where

$$ {{\omega}}_{\mathrm{n}}\equiv \sqrt{\frac{k}{J}}, \quad {\upzeta }\equiv \frac{c}{{c}_{\mathrm{c}}}, \quad {c}_{\mathrm{c}}\equiv 2\sqrt{kJ}$$

are the natural frequency, damping ratio and critical damping respectively.

Applying Laplace transform and solving for $\theta (s)$ yields

2 $$ \Theta (s)=\frac{s{\theta}_{0}}{ {s}^{2}+2\zeta {\omega}_{n}s+{\omega}_{n}^{2}}+\frac{2\zeta {\omega}_{n}{\theta}_{0}+\dot{{\theta}_{0}}}{ {s}^{2}+2\zeta {\omega}_{n}s+{\omega}_{n}^{2}}-\frac{{M}_{f}}{Js\left( {s}^{2}+2\zeta {\omega}_{n}s+{\omega}_{n}^{2}\right)}$$

By inverse Laplace transform, the time response can be obtained as

3 $$ \theta (t)=\left\{\begin{array}{c}{e}^{-\zeta{\omega}_{\mathrm{n}}t}\left(\left({\theta}_{0}+\frac{{M}_{f}}{J{\omega}_{\mathrm{n}}^{2}}\right)\mathrm{cos}\left({\omega}_{\mathrm{d}}t\right)+\left(\zeta{\omega}_{\mathrm{n}}\left({\theta}_{0}+\frac{{M}_{f}}{J{\omega}_{\mathrm{n}}^{2}}\right)+\dot{{\theta}_{0}}\right)\frac{\mathrm{sin}\left({\omega}_{\mathrm{d}}t\right)}{{\omega}_{\mathrm{d}}}\right)-\frac{{M}_{f}}{J{\omega}_{\mathrm{n}}^{2}},  \zeta <1\\ \qquad\qquad\qquad\qquad\qquad\qquad\quad{e}^{-{\omega}_{\mathrm{n}}t}\left(\left({\theta}_{0}+\frac{{M}_{f}}{J{\omega}_{\mathrm{n}}^{2}}\right)\left(1+{\omega}_{\mathrm{n}}t\right)+\dot{{\theta}_{0}}t\right)-\frac{{M}_{f}}{J{\omega}_{\mathrm{n}}^{2}},  \zeta =1\\{e}^{-\zeta{\omega}_{\mathrm{n}}t}\left(\left({\theta}_{0}+\frac{{M}_{f}}{J{\omega}_{\mathrm{n}}^{2}}\right)\mathrm{cosh}\left(\beta t\right)+\left(\zeta{\omega}_{\mathrm{n}}\left({\theta}_{0}+\frac{{M}_{f}}{J{\omega}_{\mathrm{n}}^{2}}\right)+\dot{{\theta}_{0}}\right)\frac{\mathrm{sinh}\left(\beta t\right)}{\beta}\right)-\frac{{M}_{f}}{J{\omega}_{\mathrm{n}}^{2}},  \zeta >1\end{array}\right.$$

Where

$$\begin{aligned} {\omega}_{\mathrm{d}}&\equiv {\omega}_{\mathrm{n}}\sqrt{1-{\zeta }^{2}}\\  \beta &\equiv i{{\omega}}_{\mathrm{d}}\\& ={{\omega}}_{\mathrm{n}}\sqrt{{\zeta }^{2}-1}\end{aligned}$$

**Fig. 5:**
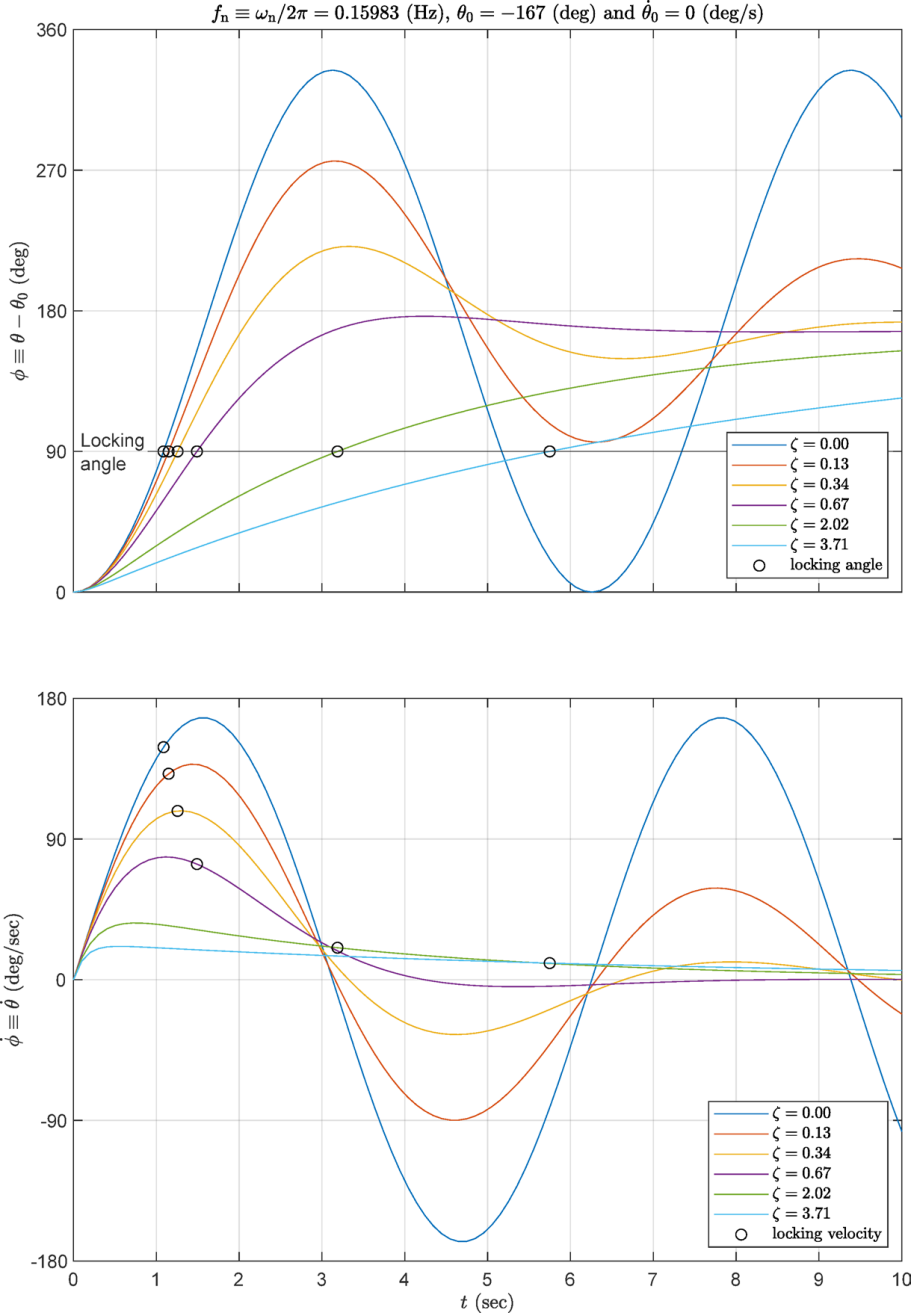
Time-history of the deployment angle and its velocity.

The derivative $$ \theta (t)$$ w.r.t. time $$ \dot{\theta}(t)$$ represents the angular velocity of the DM. For relevance Fig. 5 plots $$ \phi \equiv \theta -{\theta }_{0}$$ and $$ \dot{\phi }=\dot{\theta }$$ for $$ J=0.74 $$ kg.m^2^, $$ k=0.74$$ N.m/rad and typical on shelf dampers^1^ having damping constants $$ c=(0,\: 0.2,\:  0.5, \: 1, \: 3, \: 5.5)$$. These values correspond to the $$ \zeta $$ values displayed in the curves. These curves are plotted for $$ {\theta }_{0}=-16{7}^{^\circ }$$ and $$ \dot{{\theta }_{0}}=0$$. The locking angle at $$ \phi =9{0}^{^\circ }$$ is emphasized by a horizontal line in $$ \phi (t)$$ curve. The value of $$ J$$ is calculated from the mass of the SA, and $$ k$$ value was designed as described in^2^.

To obtain a smooth/gradual deployment, the deployment time is decided to be not less than 5 s. Table 1 shows the deployment times, versus damping constant. By examining this table, the deployment time for $$ \zeta =3.71$$ or $$ c= 5.5$$ is found as 5.7 s. By examining the $$ \dot{\phi} (t)$$ curve at this damping value, the peak angular velocity is found to be 21.3 deg/sec, and its value at the instant of locking is 10.6 deg/sec. These values show low angular momentum during deployment. That is, the designed DM yields quite satisfactory response.

**Table 1:** Deployment time velocity at instant of locking with different damping.

$$ c$$ (*N*.m/(rad/s))	Deployment time (sec.)	Angular speed (deg/s)
0	1.0	155
0.2	1.1	140
0.5	1.3	100
1	1.5	60
3	3.2	10
5.5	5.7	10

## Finite element analyses (FEA) of deployment

Comprehensive simulations on desktops can save considerable time, money and efforts. The Finite Element (FE) Method (FEM)^3^ has emerged as an indispensable tool for simulating the behavior of structures.

The FEM was initially developed to simulate simple static loads affecting structures having linear material properties and small deformations. Over the years, the FEM matured and extended to handle more complex loads, Boundary Conditions (BC’s) and material behaviors, etc. These include residual and thermal stresses, inertia, non-linear BC’s such as contacts, and non-linear inelastic materials. The FEM has been further extended to simulate the dynamics of highly nonlinear systems employing large deformation, rotation and time-varying properties.

This section simulates the dynamic response of the DM designed in Sect. 3.

### Model idealization

**Fig. 6:**
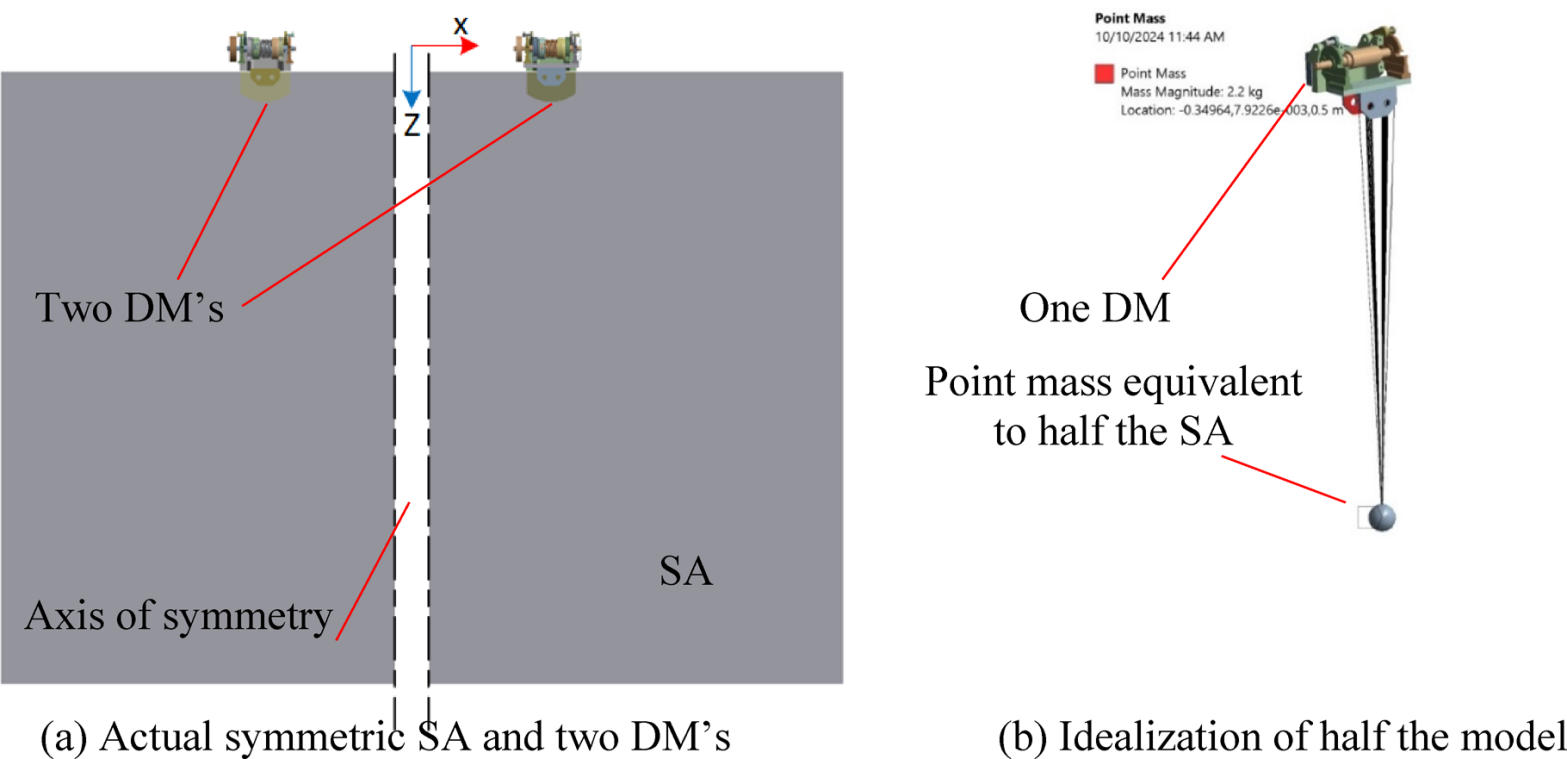
Actual and idealized models.

Figure 6-a shows the SA and the two DM’s which attach the SA to the satellite body. Due to the symmetry of the SA about Z-axis, only half the SA and one DM are simulated. This considerably reduces the computational resources and time of the FE simulation. Since the SA dynamics is beyond this study, the SA is therefore modeled as a point mass having mass and Moments of Inertia (MOI’s) equivalent to the SA, as shown in Fig. 6-b. This reduces the computational resources and time furthermore. Since half the model is idealized, the SA point mass is equivalent to half the SA. The MOI’s are calculated around the centroid of the modeled half SA.

**Fig. 7:**
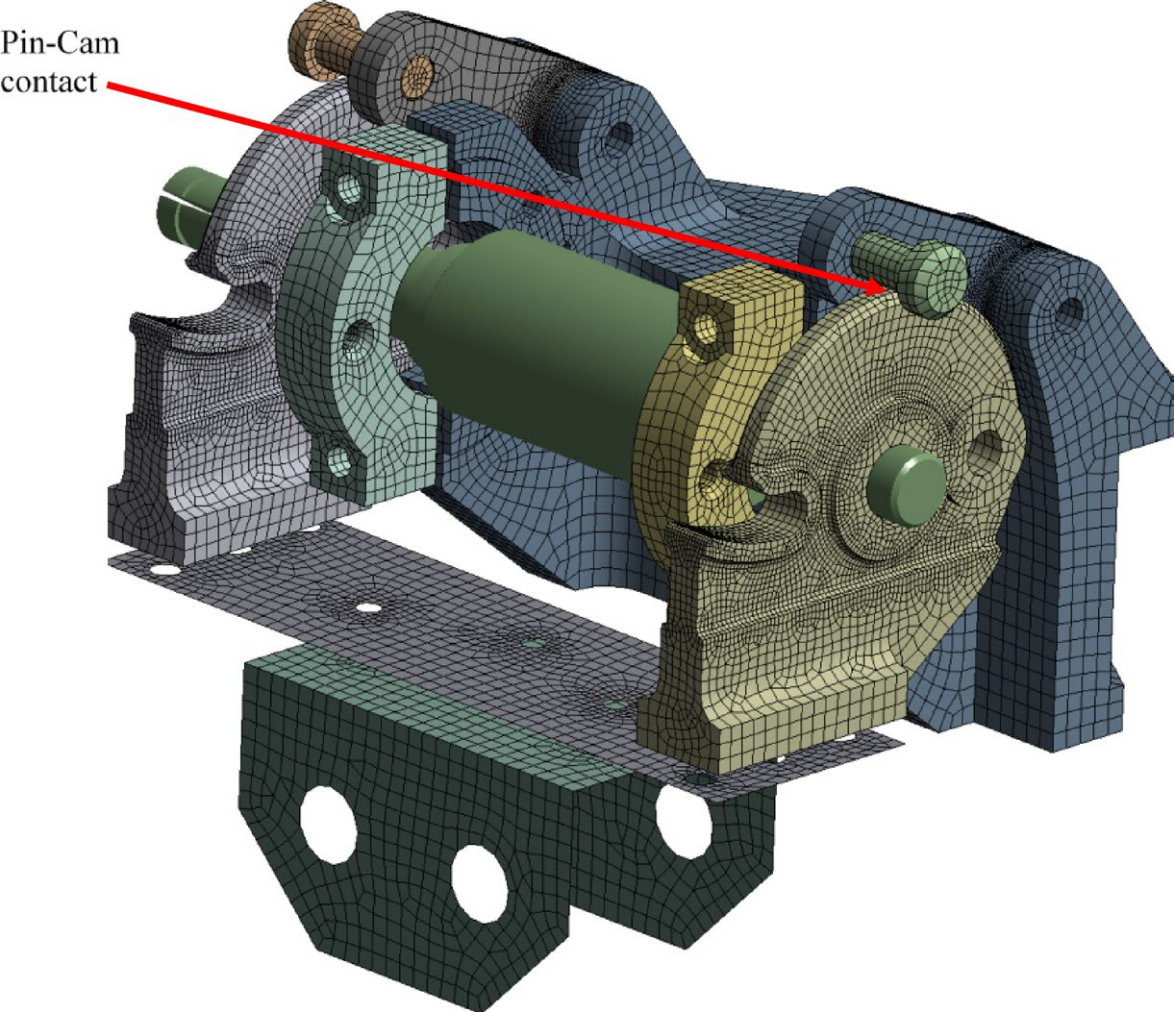
FE model of the DM.

Ansys Mechanical was used to implement the FEA of the SADM. Figure 7 shows the used mesh of the DM. As shown in the figure, for 3D elements, solid 185 and 168 are used, thin parts are modelled using quadrilateral shell elements. All bolts are modelled using 1D beam elements. The drive spring is modelled using torsional spring element Combine 14. Tiny holes and faces are cleaned to obtain a good mesh quality with adequate density, as shown in Fig. 7. The mesh density is confirmed to yield mesh independent solution. For nonlinear contact, the contact formulation is pure penalty with a friction coefficient of 0.2 and stabilization factor of 0.05.

The finite element mesh consists of a mixed-element formulation, as summarized in Table 2. Quadratic hexahedral and quadrilateral elements are used for solid and plate-like components, respectively. Beam elements model structural connections, while COMBIN14 elements represent localized stiffness and damping effects. Concentrated inertia is included using lumped point mass elements.

**Table 2:** Mesh statistics Summary.

Element Type	Ansys Name	No. of Elements	No. of Nodes
Quadratic Hexahedral	SOLID186	900 K	750 K
Quadrilateral	SHELL181		
Spring–Damper	COMBIN14		
Lumped Mass	MASS21		
Beam elements	BEAM188		

### Time stepping

**Fig. 8:**
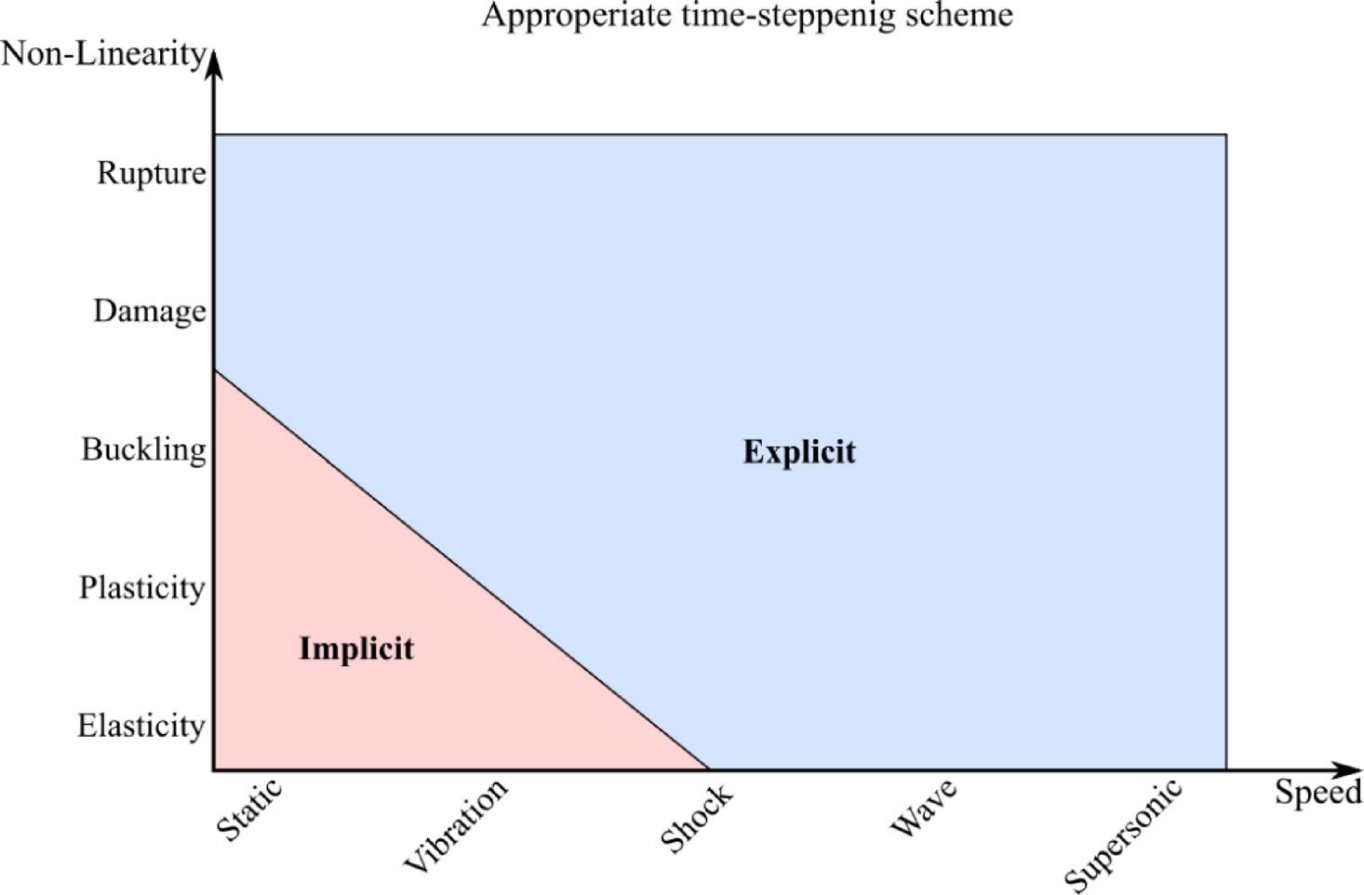
Implicit versus explicit schemes. (adapted from^4)^.

To simulate the dynamic problems versus time, time-stepping is required. Time-stepping typically uses the Finite Difference Method (FDM). Time-stepping using the backward-difference scheme is denoted “implicit”, while that using the central-difference scheme is denoted “explicit”^3^. A comparison of both schemes is presented in Fig. 8.

In the backward-difference implicit schemes, the information needed for time-stepping are implicitly available. Therefore, a coupled system of equations is to be solved for every time step, using a non–linear algorithm such as Newton–Raphson’s. Theoretically, the time step $\Delta t$ can be large due to the unconditional stability of implicit schemes. Practically however, $\Delta t$ is decided based on how easily the Newton–Raphson scheme converges. The more non-linear the problem is, $\Delta t$ can become very small making the solution time prohibitively long. This limits usage of implicit schemes for static or slow varying problems having linear or moderately nonlinear properties^4^.

In contrast, in the central-difference explicit schemes, the information needed for time-stepping are explicitly available. Therefore, the solution at the new time step is straight-forward. This enables targeting highly nonlinear problems, but however at a price. $\Delta t$ must be less than the Courant time, which is the time taken for the fastest stress wave to cross the smallest element. Accordingly, for problems with large time duration, the solution time can be prohibitively long. $\Delta t$ is however not influenced by the loading rate and/or problem non–linearity. This enables applying the explicit schemes in highly nonlinear problems involving fast transients, crash, impact, blast, wave propagation, etc. The implicit and explicit schemes were compared in^5^. Recent advancements in both explicit and implicit methods have been explored in various studies over the last decade^6–16^.

Regarding the studied SADM, due to its relatively slow deployment, the implicit time-stepping is used to simulate the deployment. However, due to the large rotations and complex contacts encountered during the deployment, the Newton-Raphson algorithm encounters great difficulties to converge. This causes $\Delta t$ to reduce to very small values. Using a constant $\Delta t$ thus making the solution time prohibitively long. Therefore, an adaptive time stepping scheme is employed to reduce $\Delta t$ only when necessary, and revert back to adequate values where the convergence is easy. The effect of damping on the convergence was also analyzed.

### Adaptive time-stepping

**Fig. 9:**
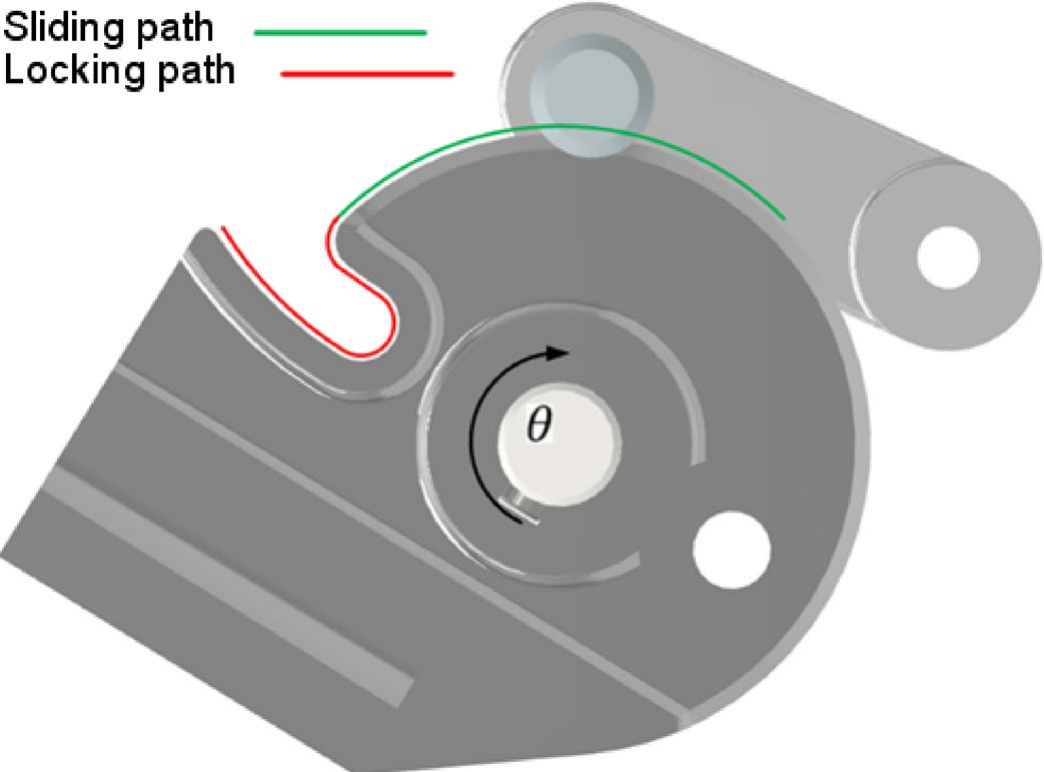
The sliding path (green) and the locking path (red).

Figure 9 shows the sliding and locking paths of the SADM. The sliding over the sliding path (cam surface) is expected to be relatively slow without considerable solution variations. Accordingly, the convergence is expected to be easy. Since the implicit schemes are unconditionally stable, a large time step $\Delta t$ can be used during this phase. On the other hand, the latching of the locking pin inside the locking grove encounters rapid variations within a very tight time duration. Accordingly, this causes convergence difficulties. To alleviate these difficulties, $\Delta t$ is reduced so that the variation within each time increment is not so rapid.

In other words, the appropriate $\Delta t$ can vary significantly throughout the solution time-history. Of course simply using a constant so small $\Delta t$ makes the solution time prohibitively long, besides wasting considerable computational cost without necessarily improving convergence. Therefore, a robust time stepping algorithm that can adaptively choose the appropriate time step $(\Delta t)$.

**Fig. 10:**
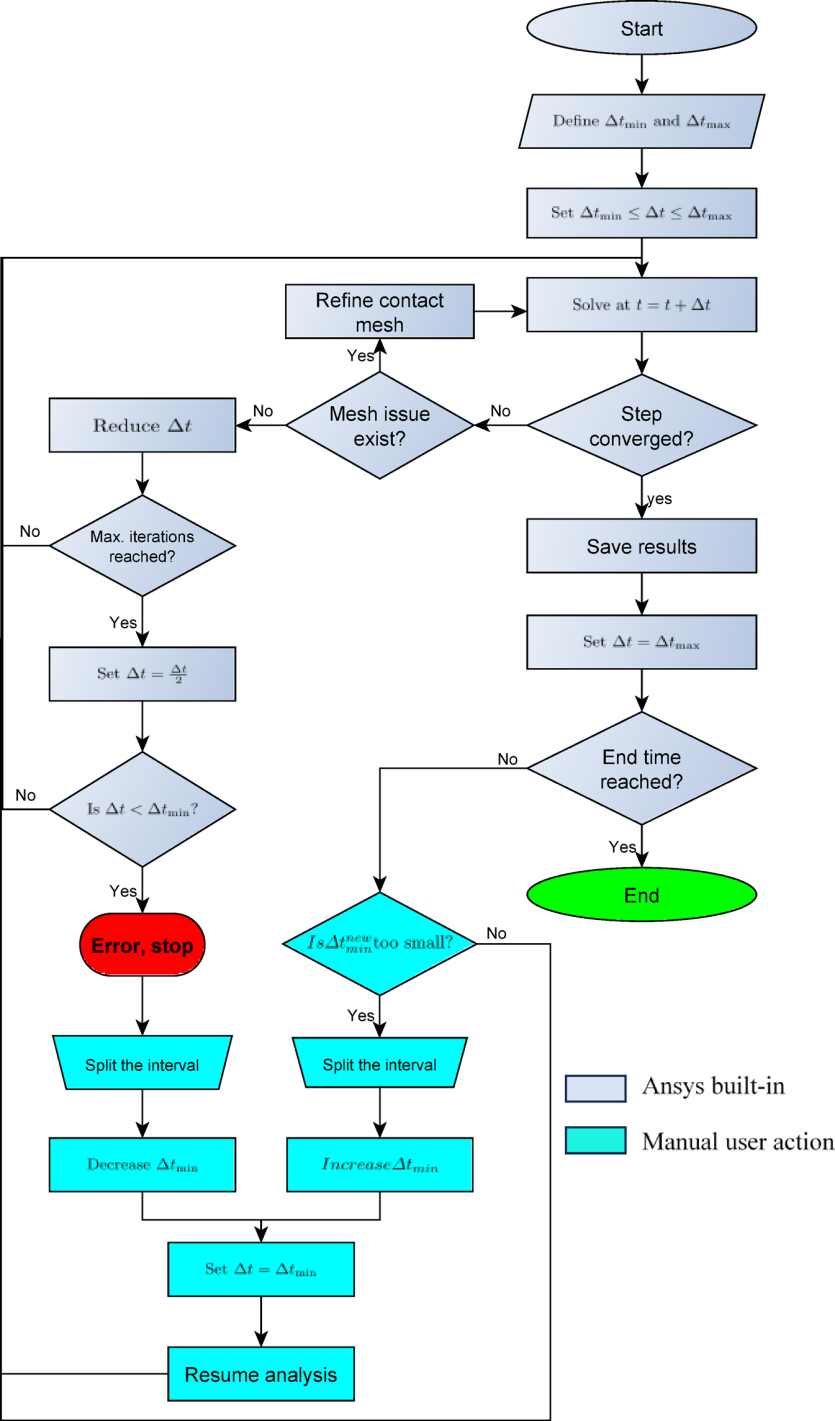
Generic adaptive time stepping and error handling algorithm.

Figure 10 shows a flowchart of an adaptive time stepping and error handling algorithm. The algorithm starts with specification of the minimum and maximum allowable time steps ${\Delta t}_{\mathrm{m}\mathrm{i}\mathrm{n}}$ and ${\Delta t}_{\mathrm{m}\mathrm{a}\mathrm{x}}$, which bounds the adaptive time-stepping algorithm. Additionally, an initial time step within this range is specified, ensuring a balance between computational efficiency and solution accuracy.

Using Newton-Raphson, or a similar, method, the solver attempts to obtain a converged solution at $t=t+\Delta t$^17^. If convergence is obtained, the simulation results are saved, and the time step is reset to ${\Delta t}_{\mathrm{m}\mathrm{a}\mathrm{x}}$ to optimize computation for subsequent steps. On the other hand, if convergence was not achieved, the algorithm examines potential problems. It first checks mesh-related issues. Poorly meshes of contact regions or bad mesh quality can lead to non-convergence. In such cases, the mesh is refined before resuming the analysis. Otherwise, $\Delta t$ size is reduced trying to obtain convergence. If convergence could not be reached by a pre-specified maximum number of iterations per step, $\Delta t$ is halved, and the solver retries finding a convergent solution again. However, if $\Delta t$ fell below ${\Delta t}_{\mathrm{m}\mathrm{i}\mathrm{n}}$, the solver terminates with an error. This can occur for highly nonlinear problems with varying properties or due to insufficiently large ${\Delta t}_{\mathrm{m}\mathrm{i}\mathrm{n}}$. In this case, the analysis time-history is split at the current instant, and ${\Delta t}_{\mathrm{m}\mathrm{i}\mathrm{n}}$ is further reduced. The solver then resumes the analysis starting with ${\Delta t=\Delta t}_{\mathrm{m}\mathrm{i}\mathrm{n}}$.

This algorithm adaptively adjusts the time step and mesh refinement as needed, reducing the computational resources to a minimum. If the convergence could not be obtained, the user should check proper boundary conditions. In this simulation, ${\Delta t}_{\mathrm{m}\mathrm{i}\mathrm{n}}=0.01$ sec. and ${\Delta t}_{\mathrm{m}\mathrm{a}\mathrm{x}}=0.05$ sec. were used. The force convergence tolerance is 0.5% with energy dissipation ratio of 1e-4.

In the first attempt to simulate the SADM, Ansys terminated the analysis as shown in the red block of the flowchart of Fig. 10. According to the chart algorithm, we split the solution interval and manually decreased the minimum time step to become ${\Delta t}_{\mathrm{m}\mathrm{i}\mathrm{n}}=0.005$ sec. After that, we split again to increase the time step when the mechanism becomes stable and no significant changes in the dynamics.

## Results and discussion

### Damping effect

**Fig. 11:**
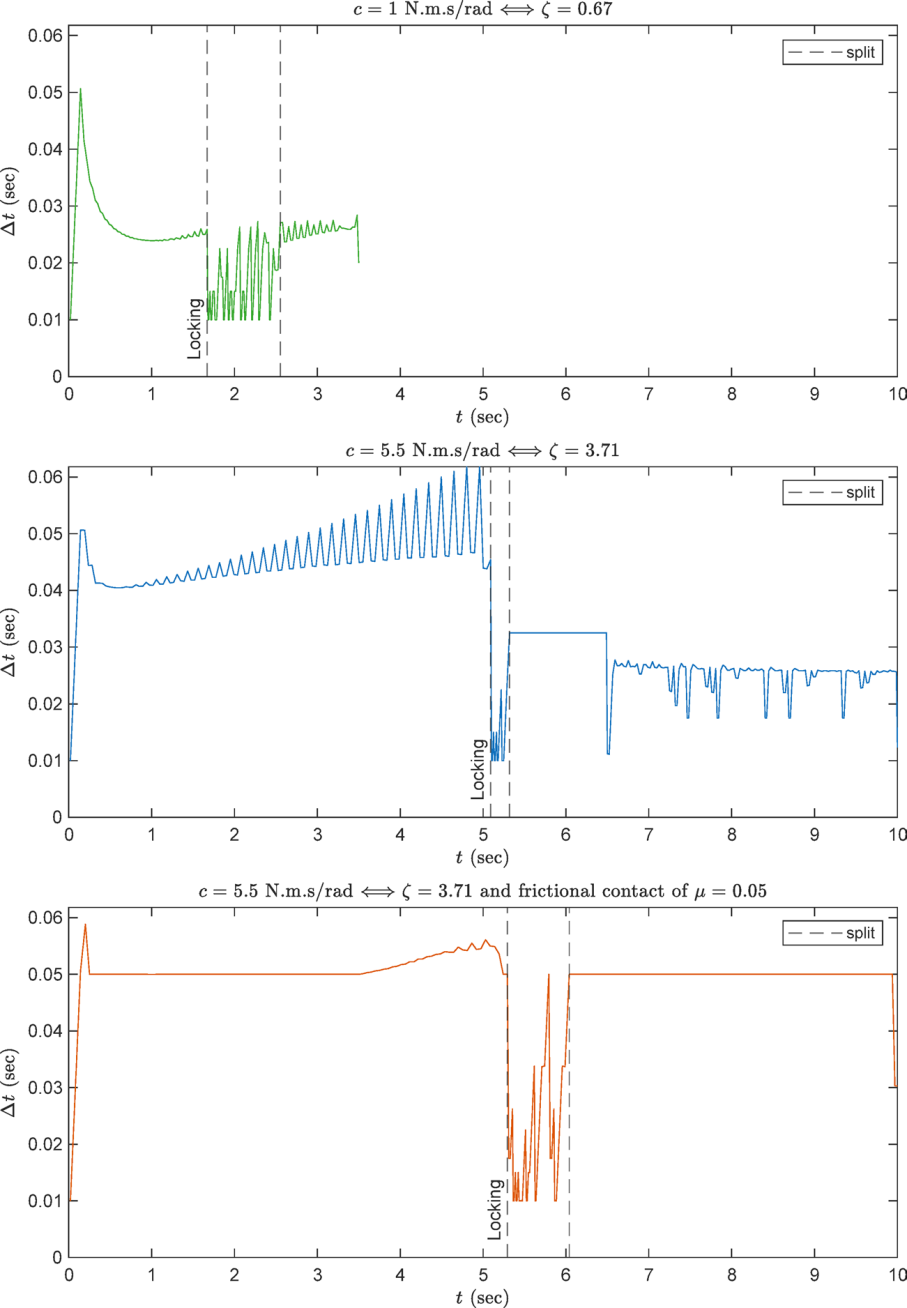
$\Delta t$ variation for several damping values.

At first, start with a damping value of $$ c=1$$ N.m.s/rad or $$ \zeta =0.67$$. As explained in the flowchart of Fig. 10, Ansys starts with the specified initial $$\Delta t$$ estimate. In the following step, the maximum specified $$\Delta t$$ value is tried. If the convergence could not be reached using this value, Ansys attempts to decrease it and try again. This behavior is shown in Fig. 11-(top) up to the instant before locking. During locking, the locking pin falls rapidly into the locking grove. This yields large deformations within a very small period of time. This dictates to a very strong geometric nonlinearity, which can make the convergence very difficult. Therefore, Ansys decreases $$\Delta t$$ to its minimum value in this phase. After the locking stabilizes, $$\Delta t$$ increases again as shown in the figure. The total computational time of this simulation is 9 h on a powerful workstation.

By using the damping chosen in Sect. 3 having $$ \zeta =3.71$$ or $$ c= 5.5$$, the deployment speed became much slower, as shown in Fig. 11(middle). Accordingly, the convergence became more easier, and hence the computational time decreased to become 5 h on the same workstation.

By adding the frictional contact between the cam and the locking pin having $\mu =0.05$, the deployment speed became a bit slower, as shown in Fig. 11(bottom). The convergence became much more easier, and hence the computational time further decreased to become 8.5 h on the same workstation.

The plots demonstrate that increasing the damping constant and introducing friction improves the nonlinear convergence behavior, reducing the number of failed substeps and promoting smoother convergence paths. Table 3 shows the statistics of the solver performance for the three-cases.

The simulations were executed on a workstation equipped with an 10th generation Intel Core i7 8 core 3.8 GHz CPU.

**Table 3:** Solver performance for various damping configurations.

Damping Cases	${\boldsymbol{t}}_{\boldsymbol{s}\boldsymbol{i}\boldsymbol{m}}\left(\boldsymbol{s}\right)$	${\boldsymbol{t}}_{\boldsymbol{s}\boldsymbol{o}\boldsymbol{l}\boldsymbol{v}\boldsymbol{e}}$	Total iterations	No. of converged iterations
Low damping	3.5	9 h 7 min	938	159
High damping, µ = 0	10	9 h 40 min	1238	244
High damping, µ = 0.2	10	8 h 30 min	1104	227

The convergence of the implicit solution improved significantly with the inclusion of damping and friction. The added damping dissipates oscillatory energy from the system, reducing high-frequency residual oscillations and stabilizing the iterative solution. Similarly, the friction moment smooths the relative motion during contact and reduces abrupt changes in the contact state. Together, these effects produce a smoother response and enable more reliable convergence, especially near the locking phase.

### Angular rotation and velocity

**Fig. 12:**
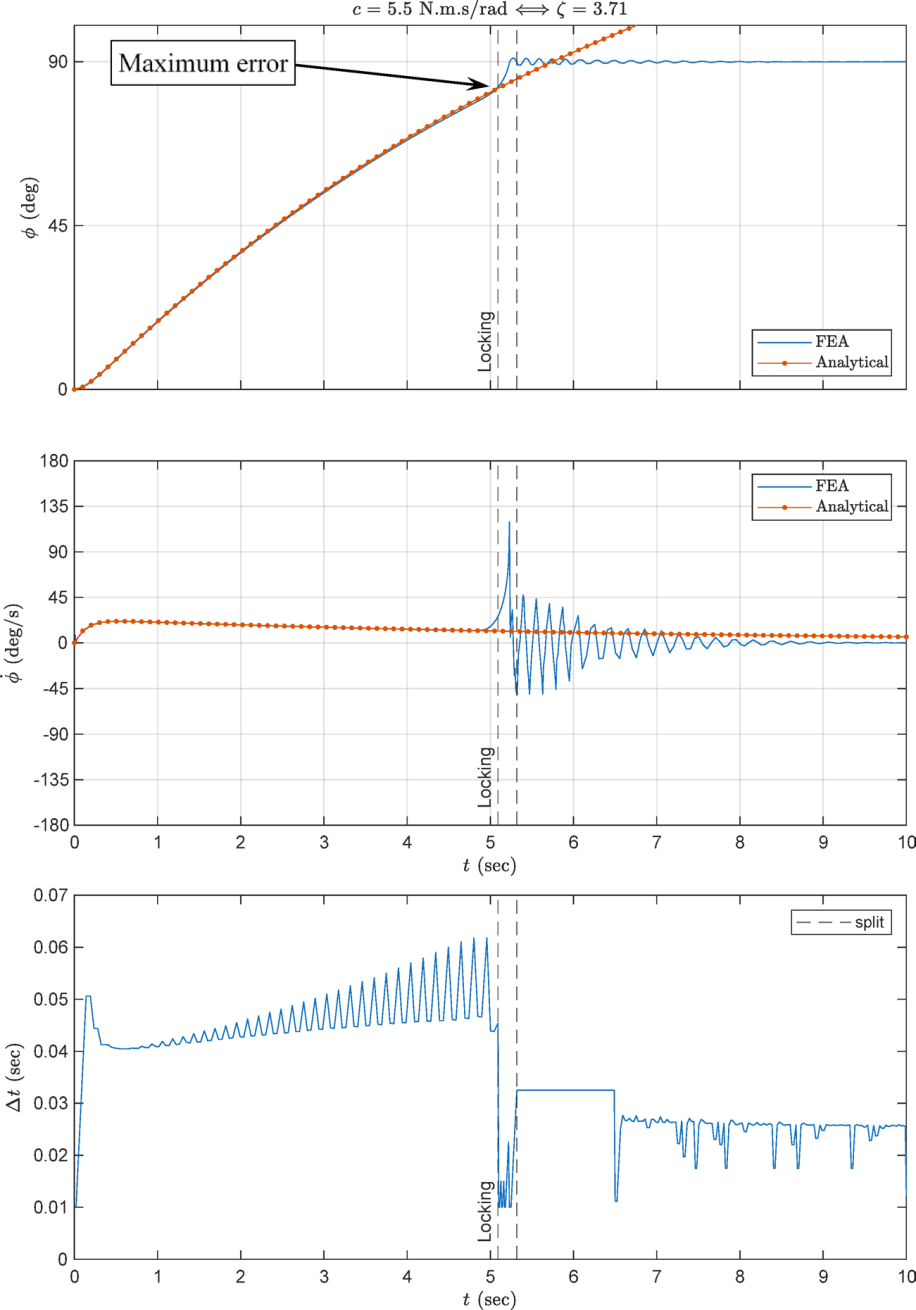
FEA verification with analytical results.

Figure 12 compares the analytical and FEA response time-histories of the angular rotation and velocity of the DM, using the damping values chosen in Sect. 3, $$ \zeta =3.71$$ or $$ c= 5.5$$. As shown in the figure, locking occurs at around 5.3 s. Both time- histories show the same trend of starting from initial condition and moving towards the locking angle with decelerating angular velocity. The analytical and FEA curves are identical up to the instant of locking with maximum relative error of 0.85%. This verifies the analytical model up to the instant of locking. After this, the analytical response is no longer valid since the locking system is not included in the analytical model. On the other hand, the FEA result shows an expected damped oscillatory behavior around the locking angle of $$ 9{0}^{^\circ }$$.

**Fig. 13:**
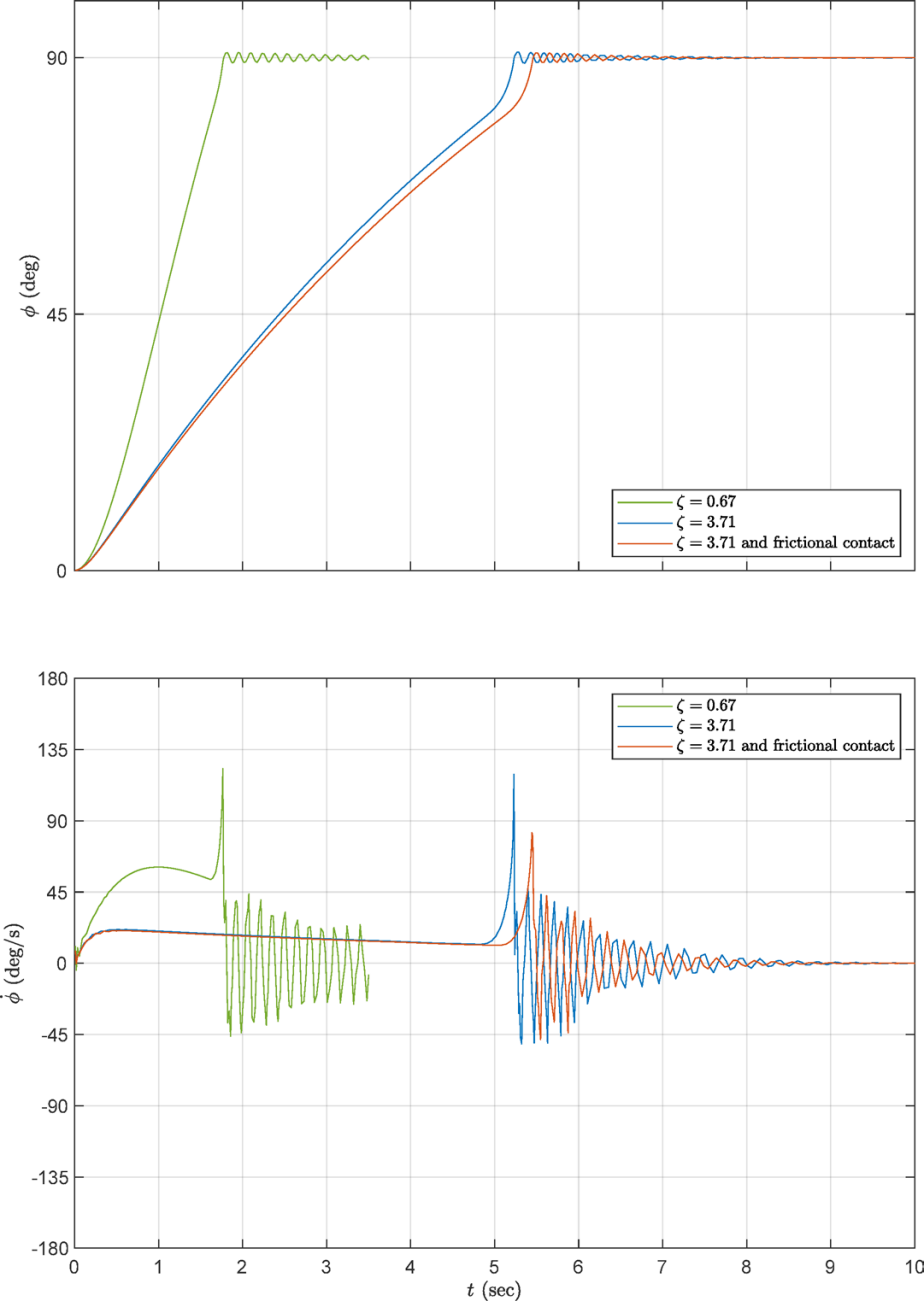
Angular rotation and velocity of the damping values of Fig. 11.

Figure 13 compares the responses of the chosen damping value of Fig. 12 with the values included in Fig. 11. As shown in this figure, the frictional contact (red curve) slightly slows down the deployment time (as compared with the blue curve), but considerably reduces the velocity locking peak, indicating better energy dissipation design. That is, including the cam frictional damping in FEA eases the convergence, reduces the computational time and yields more realistic FEA results with reduced shock. On the other hand, the low damping value of the green curve makes the deployment time more rapid, with considerably increased velocity. This is undesirable due to the consequent considerably stronger locking shock.

The FEA results of Figs. 12 and 13 prove the robustness of the adaptive time stepping algorithm of Sect. 4.3.

### Stresses

**Fig. 14:**
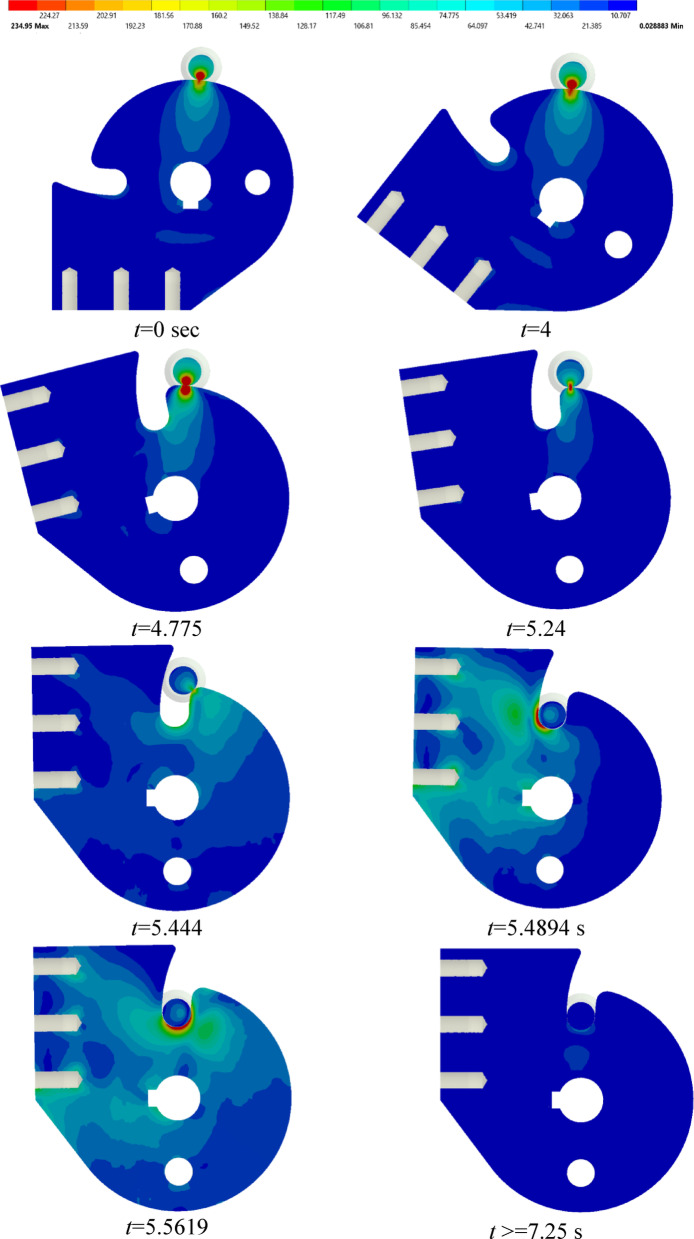
Deployment time-history and von Mises stress of the SADM.

Figure 14 shows the deployment time-history and the corresponding von-Mises stress from the stowed to locking positions. As shown, the contact stress remains constant along the sliding path, from the start till *t* = 4.775 s. Along the locking path, the stress varies considerably, from *t* = 4.775 s. In addition to the stress contour in Fig. 14, the maximum von Mises stress during locking was found to be 235 MPa, located at the locking. This value is well below the material yield strength (503 MPa), giving a safety factor of 503/235 = 2. Therefore, the locking event does not induce any risk of yielding in the mechanism.

## Conclusion

For SADM’s, locking causes shocks that can damage both the SA and the satellite structure. Therefore, a SADM was designed to decelerate the deployment before the instant of locking. This could be achieved by establishing an analytical model of the DM. Using this model, a damping constant of $\zeta =3.71$ was decided. This yielded a deployment time of 5.7 s. and acceptably low angular momentum time-history up to the instant of locking.

This design is verified using implicit transient FEA. The large rotations, complex contacts and rapid locking of the DM however make the convergence too difficult. Therefore, an adaptive time-stepping algorithm had to be employed to reduce $\Delta t$ only when necessary for convergence, and revert back to adequate $\Delta t$ values where the convergence is easy. In the studied SADM case, this algorithm could yield a successful complete analysis. It was also found that the higher the viscous damping and sliding friction, the easier the convergence of implicit FEA hence the less the computational time, as detailed in Table 2. The calculated deployment time-history showed smooth deployment with decelerating angular velocity, as designed. Locking is found to yield negligible damped oscillations and stress less than 50% of the yield stress.

This work demonstrates how implicit transient FEA can solve challenging problems having slow transients with large rotations and sudden shocks. This also extends to any relatively slow mechanism with sudden events.

Future work can address the sensitivity to manufacturing tolerances, deployment repeatability, and thermo-structural on-orbit variability.

## Data Availability

The datasets generated during the current study are owned by the Egyptian Space Agency (EgSA) and are not publicly available. Reasonable requests are to be directed to EgSA CEO office (ceo.office@egsa.gov.eg).

## Abbreviations

SADM: Solar Array Deployment Mechanism
FEA: Finite Element Analysis
ODE: Ordinary Differential Equation
$\mathrm{M}\mathrm{O}\mathrm{I},J$: Moment Of Inertia
$$ c$$: Viscous damping constant
$k$: Stiffness of the driving spring
$$T$$: Kinetic energy
$$ U$$: Elastic potential energy
$$ \theta =\varphi $$: Angular rotation
$$ \dot{\theta }=\dot{\varphi }$$: Angular velocity
${\omega}_{n}$: Angular natural frequency
$$ {N}_{conv}$$: Number of converged iterations
${\alpha}_{0}$: Angle of locking arm
$$ {N}_{total}$$: Total number of iterations
$\zeta$: Viscous damping coefficient
${\Delta t}_{\mathrm{m}\mathrm{i}\mathrm{n}}$: Minimum time step (s)
${\Delta t}_{\mathrm{m}\mathrm{a}\mathrm{x}}$: Maximum time step (s)
${\Delta t}_{i}$: Initial time step(s)
$$ {F}_{\mathrm{n}}$$: Normal force
$\mu$: Sliding friction coefficient
${M}_{\mathrm{f}}, {F}_{\mathrm{f}}$: Frictional moment, force
R: Locking arm length
$$ {t}_{sim}$$: Simulation time
$$ {t}_{solve}$$: CPU solving time
